# Preliminary Effectiveness and Safety of High Frequency Oscillation in Addition to Mechanical Insufflation and Exsufflation for Intratracheal Mucus Removal in Patients With Neuromuscular Disease: Protocol for a Prospective Study

**DOI:** 10.2196/12102

**Published:** 2019-06-27

**Authors:** Hiroyuki Awano, Masashi Nagai, Ryosuke Bo, Mariko Murao, Yusuke Ishida, Tsukasa Tanaka, Kazumi Tomioka, Masahiro Nishiyama, Hiroaki Nagase, Kazumoto Iijima

**Affiliations:** 1 Department of Pediatrics Kobe University Graduate School of Medicine Kobe Japan

**Keywords:** neuromuscular diseases, airway management

## Abstract

**Background:**

Mechanical insufflation-exsufflation (MI-E) is necessary for noninvasive management of respiratory clearance in patients with neuromuscular disorders (NMDs). Its utility has been proven, and the technique is recommended in a number of international guidelines for the management of patients with NMDs. However, the clearance of thick secretions adhering to the tracheobronchial walls could be problematic when these patients suffer from respiratory tract infections. To improve the effectiveness of the noninvasive technique, a novel device combining MI-E with high frequency oscillation (HFO) has been developed. However, the efficacy of HFO therapy in NMDs has not been well studied.

**Objective:**

The aim of this study was to elucidate the effect of MI-E combined with HFO for mucus removal in NMD patients. To evaluate its efficacy, changes in transcutaneous oxygen saturation (SpO_2_), which may predict intratracheal mucus removal, will be measured before and after use of MI-E.

**Methods:**

This is a single-center, nonblinded, nonrandomized prospective study that will enroll 5 subjects hospitalized in Kobe University Hospital owing to respiratory tract infection. All subjects will receive MI-E therapy a few times daily and will receive HFO every other day, for 6 days. Before and after MI-E use, SpO_2_ will be obtained and the change in SpO_2_ (ΔSpO_2_) between MI-E with and without HFO will be calculated. For every subject, the average of ΔSpO_2_ with or without HFO will be obtained and the null hypothesis that there is a mean change of 0 in the SpO_2_ between MI-E with and without HFO will be tested using the paired *t* test. If the treatment with HFO is found to be statistically significantly superior to the treatment without HFO, the study will conclude that HFO addition is more efficacious than no HFO addition.

**Results:**

A total of 2 subjects have already been recruited and enrolled in this study as of August 2018.

**Conclusions:**

This unique protocol will assess the efficacy of adding HFO to MI-E during the acute phase of respiratory tract infection in patients with NMDs.

**International Registered Report Identifier (IRRID):**

DERR1-10.2196/12102

## Introduction

### Background

Patients with neuromuscular disorders (NMDs) often have weak breathing muscles. As the disease progresses, vital capacity declines and the inability to expand and empty the chest fully results in reduced chest wall compliance [1-3]. As peak cough flow correlates with disease progression and lung capacity in patients with NMDs [4,5], insufficient coughing is problematic in patients with severe and advanced NMDs. Cough efficacy is related to clearance of secretions in the lung. Retention of intratracheal mucus leads to airway obstruction, causing increased work of breathing, decreased oxygenation, and ultimately respiratory failure [6]. Weak cough is thus a critical factor contributing to respiratory morbidity in NMD patients [7].

Therefore, coughing aids are necessary for patients with NMDs, and the use of assisted coughing techniques has been an important advance in the respiratory care of such patients, allowing intratracheal mucus to be controlled without the need for invasive methods, such as tracheostomy. Coughing aids include manually assisted and mechanically assisted aids [8]. Mechanical insufflation-exsufflation (MI-E) is performed using a device that generates positive pressure, provoking insufflation of the lung, and negative pressure, inducing exsufflation. A rapid change from positive to negative pressure generates air flow during exsufflation and facilitates the removal of intratracheal secretions [3,9].

It has been proven that MI-E helps to increase the peak cough flow, reduce the chance of hospitalization, permit a paradigm shift from invasive tubes to noninvasive management, and facilitate discharge to home [9-12]. Therefore, the use of MI-E is recommended in a number of international guidelines for the management of patients with NMDs [13-17].

Even if proper respiratory care is given, an inability to cough effectively and clear secretions puts patients with NMDs at risk of ventilatory failure during respiratory infection [18]. To improve the effectiveness of noninvasive techniques especially in cases of acute respiratory failure, a novel device that combines MI-E with high frequency oscillation (HFO) has been developed. During insufflation, exsufflation, or both phases, the device generates high-frequency oscillatory vibrations.

The application of HFO to the airway in patients with chronic obstructive pulmonary disease has been shown to change the viscoelastic properties of the secretions, making them more mobile [19]. Therefore, the addition of HFO to MI-E is expected to remove tenacious secretions in a noninvasive manner.

### Objectives

The aim of this study is to elucidate the effect of MI-E with HFO in terms of mucus removal in patients with NMDs. To evaluate the efficacy of the approach, changes in transcutaneous oxygen saturation (SpO_2_) and heart rate, and the subjective amount of mucus removed, are to be measured before and after use of MI-E, with and without HFO. The safety of the approach will also be evaluated by noting the frequency of adverse events and complications.

## Methods

### Study Design and Setting

This is a single-center, nonblinded, nonrandomized prospective study. As the use of oscillation is obvious, this study cannot be performed blindly. The study will be performed in the Department of Pediatrics, Kobe University Hospital, in Kobe, Japan. The study implementation period is from March 27, 2018, to January 31, 2022 (enrollment period: 3 years; follow-up period: 4 months from enrollment of the last subject).

### Eligibility Criteria

This study is conducted in patients who fulfill the following inclusion criteria and who do not meet any of the following exclusion criteria.

The inclusion criteria are as follows: patients with NMDs hospitalized in Kobe University Hospital owing to respiratory tract infection, who have already used MI-E without HFO or have started MI-E use in this study in addition to noninvasive ventilation, tracheostomy ventilation, and home oxygen therapy, and who provide written voluntary consent (or whose parents provide written voluntary consent) to participate in this study.

The exclusion criteria are as follows: patients with severe acute respiratory failure who have a very low SpO_2_ level (<90%) for more than 1 hour, albeit adequate ventilation or oxygen therapy; those who do not have tracheostomy and require intubation; those who refuse to participate in the study; and those for whom it is deemed inappropriate to participate in this study in the opinion of the investigator. These exclusion criteria are set to target patients with NMDs who have mild-to-moderate respiratory infection and to secure the safety of the subjects.

### Interventions

#### Description of Material

The Cough Assist E70 (Philips Respironics) is employed in this study. The device is used according to the manufacturer’s recommendation. The Cough Assist E70 is applied via an oronasal mask or the tracheostomy opening by trained parents, nurses, or doctors. It is used at least 3 times a day, as well as whenever SpO_2_ decreases, heart rate increases, or the patients have an increase in dyspnea or sense of retained secretions, in addition to the standard treatment, including oxygen supplementation and increased oxygen. It is set at 20 to 40 cm H_2_O for insufflation and −20 to −40 cm H_2_O for exsufflation pressure, with an insufflation and exsufflation time ratio of 1.5 to 2.5 and 0.8 to 1.5 seconds, respectively, and a pause of 0.5 to 2.0 seconds between each cycle. A total of 3 to 5 cycles are applied in every session. The frequency setting of oscillation is 15 Hz and the amplitude setting is 5 cm H_2_O. This oscillation frequency was derived from a similar previous study [20]. As a>5 cm H_2_O amplitude in the beginning of HFO use is not tolerated in child patients based on our clinical experience, 5 cm H_2_O is employed.

#### Description of the Processes and Interventions

The principal subinvestigator will obtain written informed assent or consent from patients or from their parents. After informed consent is obtained, the principal investigator or subinvestigator will determine the subject’s eligibility for enrollment. Enrollment should be done within 2 days after obtaining written consent, and protocol treatment should be started within 1 day after enrollment.

For the patients currently using MI-E, they will continue to use it with their existing settings. For new MI-E users, the setting described above will be used.

The following subject information will be recorded in the case report form (CRF) at the time of obtaining informed consent: date of informed consent; subject identification code; and subject’s baseline characteristics, including sex, birth date, height, body weight, medical history, concurrent diseases, and original setting of MI-E.

**Figure 1:**
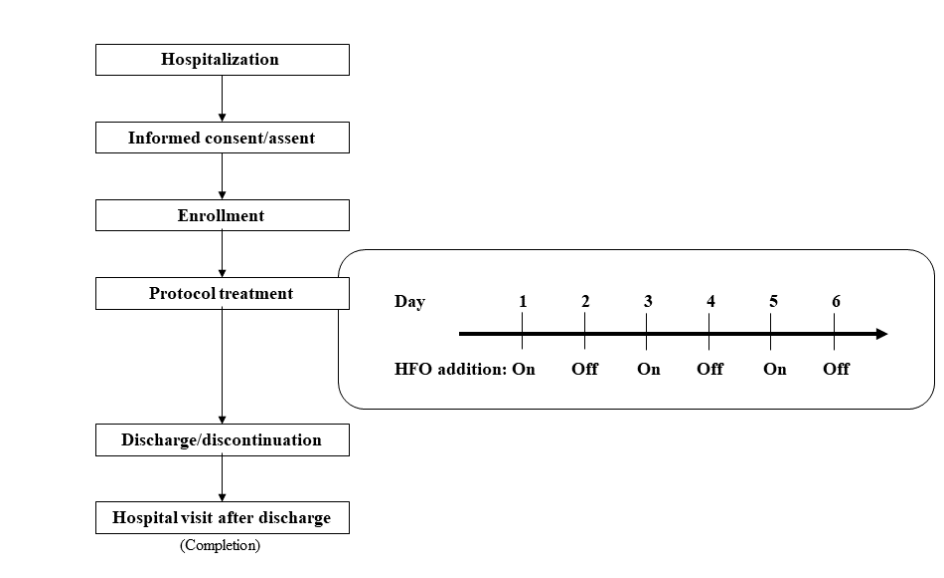
Outline of the study. Subjects will receive high frequency oscillation (HFO) every other day for 6 days. This shows the study schedule when the subject is randomized to start the protocol with HFO-on.

HFO will be added to MI-E in every subject on every other day, for 6 days. The addition of HFO on day 1 or day 2 will be determined in a random manner. When the subject is randomly selected to start the protocol with HFO on, the subject will receive MI-E with HFO treatment on days 1, 3, and 5 (Figure 1). If the subject is randomly selected to start the protocol with HFO off, they will receive MI-E with HFO on days 2, 4, and 6. The subject will thus be treated using MI-E with HFO for 3 days and without HFO for 3 days. During protocol treatment, the time for switching to HFO on and off has been set as 9 am.

#### Criteria for Discontinuation

When continuation with the study is judged to be impossible for any of the following reasons, the principal investigator or subinvestigator will terminate the subject’s participation in the study and specify the date and time of discontinuation or dropout, reason for discontinuation, and clinical course in the medical records and CRF. The reasons for discontinuation include the following: when the subject or their parents request to withdraw from the study; when the subject’s respiratory status worsens and continuation of the study treatment is judged to be undesirable; when an adverse event, including pneumothorax, occurs and further continuation with the study is difficult; when the subject is discharged from the hospital; and when discontinuation from the study is appropriate for other reasons in the opinion of the principal investigator or subinvestigator.

When subjects who have already used MI-E have completed the protocol, MI-E will be set at the original setting. For subjects who commence use of MI-E for the first time in this study, MI-E will be set at a clinically favorable setting. HFO will be added if requested by the subject. For each subject, the observation period will end on the day when the subject visits the outpatient clinic of Kobe University Hospital for follow-up at 2 to 4 weeks after discharge.

### Outcomes

#### Primary Outcomes

Oxygenation change (SpO_2_) is the primary outcome in this study. Data will be obtained at every MI-E use during the study protocol. SpO_2_ values will be recorded at any point within 15 min before MI-E treatment and at 3 min after treatment.

#### Secondary Outcomes

To evaluate the efficacy of the treatment, changes in heart rate will be obtained at the same time point used for SpO_2_. Additionally, the amount of mucus removed will be assessed subjectively, according to 3 grades (little, moderate, and abundant). As it was recommended by an external reviewer, peak cough flow was added to the secondary outcomes at the midpoint of the study. As safety end points, adverse events and the incidence of complications during the observation period will be obtained. The worst grade of the event will be considered the severity grade of each observed adverse event.

### Data Analysis Methods

#### Target Sample Size and Rationale

The primary endpoint of this study is the change in SpO_2_ (ΔSpO_2_) between MI-E with and without HFO. As no previous study has elucidated HFO efficacy in patients with NMDs, it is difficult to estimate how much change will be achieved. We assumed that the ratio of the average ΔSpO_2_ difference between HFO-on and HFO-off to the standard deviation would be about 1.1 to 1.2. To test the null hypothesis that there would be a mean change of 0 in the SpO_2_ between MI-E with and without HFO, using a paired *t* test with a 2-sided significance level of 5% and 80% power for the analysis of the primary endpoint, the required number of subjects was calculated to be 5. MI-E is applied more than 3 times a day, at least 45 data points are expected to be obtained from HFO-on or HFO-off. As the number of subjects enrolled per year can be assumed to be 1 to 2, the enrollment period has been set to 3 years.

#### Statistical Analysis

This study will evaluate weather MI-E with HFO is significantly superior to MI-E without HFO in terms of the change in SpO_2_ as the primary endpoint of this study. If the protocol treatment with HFO is found to be statistically significantly superior to the treatment without HFO, the study will conclude that the addition of HFO to MI-E is more efficacious than not adding HFO.

The ΔSpO_2_ is calculated by subtracting the SpO_2_ value of pre-MI-E from the SpO_2_ value of post-MI-E. For every subject, the average of ΔSpO_2_ with or without HFO will be obtained. Then, a statistical analysis will be performed using a paired *t* test for the average ΔSpO_2_ values. The hypothesis testing will use a 2-sided significance level of 5%, with a calculation of the 2-sided 95% CI. The change in heart rate (ΔHR) as the secondary efficacy endpoint will be examined in the same way.

The safety endpoint of this study is the frequency of adverse events and complications. A summary table will be prepared for this endpoint. For estimation of the proportion, the exact 2-sided 95% CI for binomial distribution will be calculated by group. As necessary, Fisher exact test will be used for intergroup comparison. The final analysis will be performed after data from the subjects have been obtained and locked after the end of the follow-up period. The full analysis dataset and safety analysis dataset will consist of all subjects enrolled in this study, who performed at least one protocol treatment and for whom efficacy data are available.

The statistical analysis will be performed using GraphPad PRISM 7.02 (GraphPad Software).

### Ethics

The study protocol was approved by the ethics committee of the Graduate School of Medicine, Kobe University (approval #290090).

## Results

A total of 2 subjects have already been recruited and enrolled in this study as of August 2018.

## Discussion

### Overview

This study will describe the efficacy of the addition of HFO to MI-E for the treatment of NMD patients with mild-to-moderate respiratory disease. A further goal is to provide the best selection of an airway clearance technique to prevent respiratory failure, especially when the patients have respiratory tract infection. The efficacy of HFO therapy in patients with NMDs has not been well studied to date. The efficacy of combined therapy including MI-E and HFO has been studied in patients with amyotrophic lateral sclerosis [20], but that study did not demonstrate an effect of increasing the peak cough flow. The peak cough flow is considered a key marker of the effect of airway clearance techniques. However, it is not recognized as a standard measurement, as the process, including device specification, instructions, and normal ranges, is not yet well-established [18]. In addition, it is difficult to test the peak cough flow on uncooperative pediatric patients or patients with advanced disease who only have a faint cough. In this study, we have set ΔSpO_2_ as the primary outcome for evaluating the effect of mucus removal by MI-E and HFO combination therapy. Continuous SpO_2_ monitoring is a simple noninvasive method of establishing the percentage of hemoglobin that is saturated with oxygen. It has been used for monitoring both the acute and chronic respiratory status of NMDs [14,21-23]. SpO_2_ has also been used as a measure of secretion clearance [24].

We recognized some limitations in this study. First, there is no previous report similar to this study. Therefore, it is difficult to estimate how much change will be obtained between HFO-on and HFO-off conditions and how many target samples will be sufficient to observe a statistically significant difference. Second, this is a single-center study, which limits the generalization of the findings. Third, the included subject group will be heterogenous, and the subjects are expected to demonstrate considerable variability in severity. Finally, as the optimal HFO setting in patients with NMDs has not been established [25], it is unclear if our appointed setting applied in this study will be appropriate for effective secretion removal.

### Conclusions

This is a unique protocol to assess the efficacy of adding HFO to MI-E during the acute phase of respiratory tract infection in patients with NMDs. This is a preliminary study in an area that requires further investigation; the findings of this study may provide a basis for developing the best way to use MI-E.

## Abbreviations

CRF: case report form
HFO: high frequency oscillation
MI-E: mechanical insufflation-exsufflation
NMD: neuromuscular disorders
SpO_2_: transcutaneous oxygen saturation

## References

[1] Inkley SR, Oldenburg FC, Vignos PJ (1974). Pulmonary function in Duchenne muscular dystrophy related to stage of disease. Am J Med.

[2] Chua K, Tan CY, Chen Z, Wong HK, Lee EH, Tay SK, Ong HT, Goh DY, Hui JH (2016). Long-term follow-up of pulmonary function and scoliosis in patients with Duchenne's muscular dystrophy and spinal muscular atrophy. J Pediatr Orthop.

[3] Fauroux B, Guillemot N, Aubertin G, Nathan N, Labit A, Clément Annick, Lofaso F (2008). Physiologic benefits of mechanical insufflation-exsufflation in children with neuromuscular diseases. Chest.

[4] Ripamonti E, D'Angelo G (2018). Measurement of respiratory function decline in patients with Duchenne muscular dystrophy: a conjoint analysis. Neurodegener Dis Manag.

[5] Ronzani F, Trivella A, Arzoumanian E, Blanc S, Sarakha M, Richard C, Oliveros E, Lacombe S (2013). Comparison of the photophysical properties of three phenothiazine derivatives: transient detection and singlet oxygen production. Photochem Photobiol Sci.

[6] Homnick DN (2007). Mechanical insufflation-exsufflation for airway mucus clearance. Respir Care.

[7] Boitano LJ (2006). Management of airway clearance in neuromuscular disease. Respir Care.

[8] Bach JR (1994). Update and perspective on noninvasive respiratory muscle aids. Part 2: The expiratory aids. Chest.

[9] Chatwin M, Bush A, Simonds AK (2011). Outcome of goal-directed non-invasive ventilation and mechanical insufflation/exsufflation in spinal muscular atrophy type I. Arch Dis Child.

[10] Bach JR, Saporito LR, Shah HR, Sinquee D (2014). Decanulation of patients with severe respiratory muscle insufficiency: efficacy of mechanical insufflation-exsufflation. J Rehabil Med.

[11] Chatwin M, Ross E, Hart N, Nickol AH, Polkey MI, Simonds AK (2003). Cough augmentation with mechanical insufflation/exsufflation in patients with neuromuscular weakness. Eur Respir J.

[12] Tzeng AC, Bach JR (2000). Prevention of pulmonary morbidity for patients with neuromuscular disease. Chest.

[13] Finder JD (2010). Airway clearance modalities in neuromuscular disease. Paediatr Respir Rev.

[14] Birnkrant DJ, Bushby K, Bann CM, Alman BA, Apkon SD, Blackwell A, Case LE, Cripe L, Hadjiyannakis S, Olson AK, Sheehan DW, Bolen J, Weber DR, Ward LM, DMD Care Considerations Working Group (2018). Diagnosis and management of Duchenne muscular dystrophy, part 2: respiratory, cardiac, bone health, and orthopaedic management. Lancet Neurol.

[15] Finkel RS, Mercuri E, Meyer OH, Simonds AK, Schroth MK, Graham RJ, Kirschner J, Iannaccone ST, Crawford TO, Woods S, Muntoni F, Wirth B, Montes J, Main M, Mazzone ES, Vitale M, Snyder B, Quijano-Roy S, Bertini E, Davis RH, Qian Y, Sejersen T, Care group SMA (2018). Diagnosis and management of spinal muscular atrophy: Part 2: Pulmonary and acute care; medications, supplements and immunizations; other organ systems; and ethics. Neuromuscul Disord.

[16] Strickland SL, Rubin BK, Drescher GS, Haas CF, O'Malley CA, Volsko TA, Branson RD, Hess DR, American Association for Respiratory Care‚ Irving‚ Texas (2013). AARC clinical practice guideline: effectiveness of nonpharmacologic airway clearance therapies in hospitalized patients. Respir Care.

[17] Hull J, Aniapravan R, Chan E, Chatwin M, Forton J, Gallagher J, Gibson N, Gordon J, Hughes I, McCulloch R, Russell RR, Simonds A (2012). British Thoracic Society guideline for respiratory management of children with neuromuscular weakness. Thorax.

[18] Toussaint M, Chatwin M, Gonzales J, Berlowitz DJ, ENMC Respiratory Therapy Consortium (2018). 228th ENMC International Workshop:: airway clearance techniques in neuromuscular disorders Naarden, The Netherlands, 3-5 March, 2017. Neuromuscul Disord.

[19] Ragavan AJ, Evrensel CA, Krumpe P (2010). Interactions of airflow oscillation, tracheal inclination, and mucus elasticity significantly improve simulated cough clearance. Chest.

[20] Sancho J, Bures E, de La Asunción S, Servera E (2016). Effect of high-frequency oscillations on cough peak flows generated by mechanical in-exsufflation in medically stable subjects with amyotrophic lateral sclerosis. Respir Care.

[21] Labanowski M, Schmidt-Nowara W, Guilleminault C (1996). Sleep and neuromuscular disease: frequency of sleep-disordered breathing in a neuromuscular disease clinic population. Neurology.

[22] Bach JR, Gonçalves MR, Hamdani I, Winck JC (2010). Extubation of patients with neuromuscular weakness: a new management paradigm. Chest.

[23] Bach JR, Baird JS, Plosky D, Navado J, Weaver B (2002). Spinal muscular atrophy type 1: management and outcomes. Pediatr Pulmonol.

[24] Chatwin M, Simonds AK (2009). The addition of mechanical insufflation/exsufflation shortens airway-clearance sessions in neuromuscular patients with chest infection. Respir Care.

[25] Chatwin M, Toussaint M, Gonçalves MR, Sheers N, Mellies U, Gonzales-Bermejo J, Sancho J, Fauroux B, Andersen T, Hov B, Nygren-Bonnier M, Lacombe M, Pernet K, Kampelmacher M, Devaux C, Kinnett K, Sheehan D, Rao F, Villanova M, Berlowitz D, Morrow BM (2018). Airway clearance techniques in neuromuscular disorders: a state of the art review. Respir Med.

